# Food-Approach Eating Behaviors and Brain Morphology: The Generation R Study

**DOI:** 10.3389/fnut.2022.846148

**Published:** 2022-04-04

**Authors:** Olga Dmitrichenko, Yuchan Mou, Trudy Voortman, Tonya White, Pauline W. Jansen

**Affiliations:** ^1^Department of Epidemiology, Erasmus University Medical Center, Rotterdam, Netherlands; ^2^The Generation R Study Group, Erasmus University Medical Center, Rotterdam, Netherlands; ^3^Institute for Medical Information Processing, Biometry and Epidemiology (IBE), Ludwig-Maximilians-Universität (LMU) Munich, Munich, Germany; ^4^Pettenkofer School of Public Health, Munich, Germany; ^5^Division of Human Nutrition and Health, Wageningen University & Research, Wageningen, Netherlands; ^6^Department of Child and Adolescent Psychiatry/Psychology, Erasmus University Medical Center, Rotterdam, Netherlands; ^7^Department of Radiology and Nuclear Medicine, Erasmus University Medical Center, Rotterdam, Netherlands; ^8^Department of Psychology, Education and Child Studies, Erasmus University Rotterdam, Rotterdam, Netherlands

**Keywords:** eating behaviors, food-approach behaviors, binge eating, adolescents, neuroimaging

## Abstract

Food-approach eating behaviors are associated with an increased risk of developing overweight/obesity and binge-eating disorder, while obesity and binge-eating disorder have also been linked with altered brain morphology in adults. To understand these associations, we examined the association of food-approach eating behaviors during childhood with adolescents' brain morphology. The sample included 1,781 adolescents with assessments of eating behaviors at ages 4 and 10 years and brain imaging data at 13 years from a large, population-based cohort. Food approach eating behaviors (enjoyment of food, emotional overeating, and food responsiveness) were assessed using the Child Eating Behavior Questionnaire. Additionally, we assessed binge eating symptoms using two items from the Development and Well-Being Assessment at 13 years of age. Adolescents participated in an MRI procedure and measures of brain morphology, including cerebral white, cerebral gray and subcortical gray matter volumes, were extracted from T1-weighted images processed using FreeSurfer. Enjoyment of food and food responsiveness at the age of 4 and 10 years were positively associated with cerebral white matter and subcortical gray matter volumes at age 13 years (e.g., enjoyment of food at 4 years and cerebral white matter: β = 2.73, 95% CI 0.51, 4.91). Enjoyment of food and food responsiveness at 4 years of age, but not at 10 years, were associated with a larger cerebral gray matter volume at 13 years of age (e.g., enjoyment of food at 4 years: β = 0.24, 95% CI 0.03, 0.45). No statistically significant associations were found for emotional overeating at both ages and brain measurements at 13 years of age. *post-hoc* analyses showed no associations of food-approach eating behaviors with amygdala or hippocampus. Lastly, we did not observe significant associations of binge-eating symptoms with global brain measurements and a priori-defined regions of interest, including the right frontal operculum, insular and orbitofrontal cortex. Our findings support an association between food-approach eating behaviors, especially enjoyment of food and food responsiveness, and brain morphology in adolescence. Our findings add important knowledge to previous studies that were mostly conducted in adults, by suggesting that the eating behavior-brain link may be visible earlier in life. Further research is needed to determine causality.

## Introduction

Obesity and eating disorders are a serious public health concern due to the rising prevalence and effects on physical health (1–3). Eating behaviors in childhood tend to have a long-term impact on eating habits and weight in adolescence and adulthood (4–7). Moreover, a healthy and balanced eating behavior established early in childhood is important for children's physical and mental health, as well as for optimal brain development (7, 8).

Food-approach eating behaviors describe one's general appetite and desire to eat. Most studies in children distinguish between emotional overeating, enjoyment of food and food responsiveness (9, 10). These food-approach eating behaviors have the potential to induce a faster eating rate and eating in the absence of hunger, which may lead to a higher caloric intake and the intake of energy-dense food of low nutritional quality (11, 12). Emotional overeating describes eating that is driven by one's emotional state, especially negative feelings. Food responsiveness reflects the amount of attention toward external food cues, whereas enjoyment of food captures the extent to which eating is experienced as pleasurable, which leads to a desire to eat. Food-approach behaviors are in part driven by neurocognitive functions, such as impulsiveness and poor emotion regulation skills (13, 14). Yet, eating behaviors may also affect these functions, as dietary intake affects brain development (15).

Research regarding the link between food-approach behaviors and brain morphology in children and adolescents is scarce, while emerging evidence in adults has shown associations of weight status and binge-type eating disorders with brain volumetric changes. For example, overweight in adults has been linked to global structural brain differences, such as gray matter volume reduction, smaller hippocampal volume and decreased white matter integrity (16–21). Likewise, frontal and corticolimbic volume, which are the regions that are involved in regulation of food desire and termination of food intake, were inversely associated with BMI (20, 22). Moreover, cross-sectional studies observed that adults with binge eating disorders showed smaller volumes in subcortical and cerebral brain areas (23, 24), and an enlarged insular cortex (25, 26). As food-approach behaviors are correlates of binge-type eating disorders and of overweight/obesity (27), it is important to investigate if the aforementioned brain volumetric changes are also associated with food-approach eating behaviors on a continuous scale in the general population. In addition, food-approach eating behaviors have been found to be relatively stable yet increasing across childhood in some children (40), which marks the need to assess repeated measurements of these behaviors in relation with brain morphology, in order to determine if there are any timing effects.

Within this context, we aimed to investigate the association between food-approach eating behaviors in early (4 years-of-age) and mid-childhood (10 years-of-age) and brain morphology in adolescents at 13 years of age in a large population-based study. We further examined the cross-sectional association between frequency of binge eating events and brain morphology. In the period from mid-childhood to pre-adolescence, food-approach eating behaviors are likely influenced by growth spurts and the onset of puberty. Therefore, assessing eating behaviors in both early and mid-childhood can validate the robustness of the association and examine whether the association appears in early life. Because of limited prior knowledge on the association between food-approach eating behaviors and brain morphology, we first assessed global brain volumetric measures. For binge eating disorder, we examined global brain volumes and specific regions of interest; including the insular cortex, orbitofrontal cortex and right frontal operculum. These regions were selected based on their function coupled with previous literature in adult patients with binge eating disorders (20, 25, 26).

We hypothesized that emotional overeating, food responsiveness and enjoyment of food in childhood would be associated with smaller cerebral gray matter, cerebral white matter, and subcortical gray matter volumes in adolescence. Further, we hypothesized that frequent binge-type symptoms, a more clinical characteristic of the food-approach eating behaviors, would be associated with a decrease in global brain volumes, coupled with decreases in the right frontal operculum, and insular and orbitofrontal cortex.

## Methods

### Study Design

This study was embedded in the Generation R Study, a population-based cohort from fetal life onwards (28). The Generation R Study was designed to identify early biological, environmental, and social determinants of growth, development, and health. Pregnant women living in Rotterdam, the Netherlands, with an expected delivery date between April 2002 and January 2006 were invited to participate. Assessments included biological samples, physical examinations and questionnaires. Written informed consent was obtained from all participating children and their parents. This study was approved by the Medical Ethical Committee of the Erasmus University Medical Center, Rotterdam.

### Study Population

Full consent for the postnatal phase of the Generation R Study was obtained for 6,625 children. For 5,535 children (response rate: 84%), we had data on eating behavior at 4 or 10 years of age. From this group, we excluded children who did not provide consent for the MRI scans at 13 years of age (*n* = 2,743), whose MRI scans could not be reconstructed using FreeSurfer (*n* = 1,003), or who had major incidental findings (*n* = 9). In total, 1,781 children (53.6% girls) with eating behavior assessed at ages 4 or 10 years and structural neuroimaging at 13 years of age were included as the study population (Supplementary Figure 1).

### Measures

#### Food-Approach Eating Behaviors

Food-approach eating behaviors were assessed using the same questionnaire when children were aged 4 and 10 years. At both ages, mothers reported on their children's eating behaviors using the Child Eating Behavior Questionnaire (CEBQ) (10). The CEBQ consists of 35 items that measure parental perceptions of a child's eating behavior using a 5-point Likert-scale ranging from “never” to “always”. Three subscales were used in the current study: emotional overeating, food responsiveness and enjoyment of food. The scale for emotional overeating consists of 4 items (e.g., “My child eats more when he/she is upset”), food responsiveness is a 5-item subscale (e.g., “Given the choice, my child would eat most of the time”) and enjoyment of food is evaluated in a 4-item subscale (e.g., “My child loves food”). Per subscale, maximum 25% missing items were allowed. The mean item score was calculated by summing the items and dividing them by the number of items of that scale that were filled out. Previously, the CEBQ has shown to have good test-retest reliability and internal consistency (10, 12, 29, 30). The reliability of the subscales was high (at 4 years, emotional overeating α = 0.53, food responsiveness α = 0.81, enjoyment of food α = 0.64; at 10 years, emotional overeating α = 0.66, food responsiveness α = 0.85, enjoyment of food α = 0.62).

#### Binge-Eating Symptoms

The frequency of binge eating symptoms, i.e., overeating and loss of control, was assessed at the age of 13 years by a self-report questionnaire that is based on the Development and Well-Being Assessment (DAWBA), an instrument that is designed to generate ICD-10 and DSM-IV or DSM-5 psychiatric diagnoses (31). Participants are asked a “yes/no” question regarding an overeating episode: “Sometimes people eat a very large amount of food in a very short time. For example, they may open the fridge and eat as much as they can find, eating and eating until they feel physically ill. This usually happens when people are by themselves; does this happen to you?” If participants responded with “yes”, they were asked how often the situation happened on average in the past 3 months with four response options ranging from “Did not happen” to “Once or more per week”.

Loss of control over eating was measured by asking all participants the following “yes/no” question: “In the last 3 months, has there been a time when you were eating and it felt like you couldn't stop? During which you just kept eating and eating and couldn't stop even if you wanted to?” If participants replied with “yes”, they were asked how often this happened on average in the past 3 months. Again, responses ranged from “Did not happen” to “Once or more per week”. Based on these questions, we created a binary variable representing frequently occurring binge-eating symptoms. If the participant answered that an overeating episode and loss of control over food happened less than once per month or never, the participant was considered as having no binge-eating symptoms. In the case that one or both behaviors were experienced once or more times per month, the participant was characterized as having frequent binge-eating symptoms.

#### Brain Measurements

Brain imaging measures were collected using high resolution T1 weighted MRI [see White et al. (32) for an overview of the sequences and imaging protocol]. Volumetric segmentation was performed with the FreeSurfer image analysis suite 5.1 (http://surfer.nmr.mgh.harvard.edu) (33). Quality assessment was performed in two steps. First, all T1-weighted scans were rated on a six-item scale for quality (unusable, poor, fairly good, good, very good, excellent). Scans rated below “fairly good” were excluded. After processing through FreeSurfer (https://surfer.nmr.mgh.harvard.edu/), all images were visually inspected for segmentation quality. FreeSurfer morphometric procedures have been demonstrated to show good test–retest reliability across scanner manufacturers and across field strengths (34). Global brain volumes (cerebral white, cerebral gray, and subcortical gray matter volume) and specific regions of interest (insular cortical, orbitofrontal cortical, and right frontal operculum cortical volumes) were used in the current study.

#### Covariates

Several covariates were considered as confounders based on previous literature. Age at the time of assessments was calculated using the child's date of birth. Sex was obtained from medical records filled in by obstetricians and midwives after birth. Child national origin was determined based on the country of birth of both parents. This was assessed using a prenatal questionnaire and subdivided in Dutch, Other Western and Non-western. Maternal educational level was reported at the time of enrolment in the study and was divided into three groups: low (no education finished and primary education finished), middle (secondary school or lower vocational training) and high (higher vocational training or University degree). Further, the household income was categorized into three groups: <1,200€, 1,200–2,200€ and >2,200€ per month. Exposure to tobacco during pregnancy was assessed prenatally and formed into three groups: never smoked during pregnancy, smoked until pregnancy was known or continued to smoke during pregnancy. Mothers reported on their psychopathology symptoms using a validated Brief Symptom Inventory (BSI) during their pregnancy in their second trimester (35). At the 6 years' visit, the child's height and weight were measured at the research center from which we calculated BMI. At the age of 8 years, dietary intake of the children was assessed with a food-frequency questionnaire from which we calculated energy intake and diet quality as described elsewhere (36). The intracranial volume of the participant's brain was assessed during the MRI visit at 13 years, simultaneously with the other brain measurements, as the regional volumes scale with head size (37, 38).

### Statistical Analysis

Sample characteristics were described as mean and standard deviation (SD) for continuous variables with normal distributions, median and inter-quartile range (IQR) for continuous variables with skewed distributions, and percentages for categorical variables. Correlations between the three different food-approach eating behaviors at ages 4 and 10 years, and binge eating symptoms at age 13 years were examined using Pearson's correlation coefficients.

Our primary analyses were conducted using multiple linear regressions to examine the associations between food-approach eating behaviors and global brain volumes (cerebral white matter, cerebral gray matter, and subcortical gray matter volume), and to examine the association of the presence of binge eating symptoms with global brain volumes and regions of interest. We used two models to explore each association. Model 1 was adjusted for child sex and age at the MRI measurement. Model 2 was additionally adjusted for maternal education, household income, child national origin, maternal smoking during pregnancy and maternal prenatal symptoms of psychopathology. For the subcortical gray matter volume outcome, we additionally adjusted model 2 for intracranial volume. *Post-hoc* analyses were performed for hippocampal and amygdala volumes if the association between food-approach eating behaviors and subcortical gray matter volume was statistically significant. In additional analyses, we tested whether associations were independent of child diet quality and BMI, by additionally adding diet quality at 8 years and BMI at 6 years to Model 2.

Missing values on covariates were imputed using multiple imputations by generating five datasets with five iterations using the Markov Chain Monte Carlo method. Pooled estimates were reported as results. Statistical significance was defined as two-sided α <0.05. We applied the Benjamini-Hochberg procedure with a false discovery rate (FDR) of 0.05 to minimize false-positive findings due to multiple comparisons (39). All statistical analyses were performed using R version 4.0.1 (R Foundation for Statistical Computing, Vienna, Austria).

## Results

Table 1 shows the characteristics of 1,781 adolescents and their mothers. The majority of the adolescents had a Dutch background (64.9%), and most mothers were highly educated (65.5%), had a net household income > 2,200€/month (equivalent to >US$2,522/month) (52.5%), and never smoked during pregnancy (69.5%). At age 13 years, 5.7% children had frequent binge eating symptoms (overeating or loss of control one or more times per month) as reported by themselves (Supplementary Table 1). Moderate positive correlations (r ranging from 0.32 to 0.48) were found among the eating behavior scales at age 4 years and their corresponding scales at 10 years.

**Table 1:** Characteristics of the study population.

	**% or mean (SD)**
**Adolescent characteristics**	
Sex, % girls	53.6
Adolescent national origin	
Dutch	64.9
Other western	9.6
Non-western	24.6
Missing	0.8
BMI at 6 years	16.0 (1.6)
Diet quality score at 8 years	4.5 (1.2)
Age at MRI scan	14.0 (0.6)
**Maternal characteristics**	
Maternal education level	
Low (High school, lower vocational education or less)	34.5
High (Higher vocational education and university)	65.5
Missing	11.2
Household income per month	
<1,200€	9.4
1,200–2,200€	18.1
>2,200€	52.5
Missing	17.2
Smoking during pregnancy	
Never	69.5
Until pregnancy was known	7.9
Continued during pregnancy	11.7
Missing	10.9

*Values are percentages for categorical variables and mean (SD) for continuous normally distributed variables*.

Table 2 shows the associations between food-approach eating behaviors and brain volumes. Enjoyment of food and food responsiveness at ages 4 and 10 years were positively associated with larger cerebral white matter, cerebral gray matter and subcortical gray matter volume. After multiple testing correction, higher scores of enjoyment of food and food responsiveness at 4 and 10 years were associated with a larger cerebral white matter volume (e.g., for enjoyment of food at age 4 years: β = 2.73, 95% CI 0.51, 4.91) and a larger subcortical gray matter volume (e.g., for enjoyment of food at age 4 years: β = 0.24, 95% CI 0.03, 0.45) at 13 years. Enjoyment of food and food responsiveness at 4 years of age, but not at 10 years, were also associated with a larger cerebral gray matter volume at 13 years. No associations were observed between emotional overeating and brain volumes. In addition, *post-hoc* analyses showed none of the three food-approach eating behaviors at 4 or 10 years was associated with amygdala or hippocampal volumes (Supplementary Table 2).

**Table 2:** Associations between food-approach eating behaviors at ages 4 and 10 years with brain volumes at age 13 years.

**Food-approach eating behaviors (per SD)**	**Model 1** **β (95%CI)**	***p***	**Model 2** **β (95%CI)**	***p***
**Cerebral gray matter volume at 13 years (cm^**3**^)**				
**At 4 years**				
Emotional overeating	−1.56 (−4.00, 0.89)	0.21	−0.02 (−2.41, 2.36)	0.98
Enjoyment of food	3.68 (1.24, 6.13)	0.003	**3.09 (0.74, 5.45)**	**0.01**
Food responsiveness	1.76 (−0.67, 4.20)	0.16	**3.16 (0.82, 5.51)**	**0.01**
**At 10 years**				
Emotional overeating	−0.34 (−2.74, 2.07)	0.78	0.91 (−1.4, 3.21)	0.44
Enjoyment of food	3.15 (0.78, 5.52)	0.01	1.94 (−0.32, 4.24)	0.09
Food responsiveness	0.27 (−2.11, 2.64)	0.83	1.03 (−1.25, 3.31)	0.37
**Cerebral white matter volume at 13 years (cm^**3**^)**				
**At 4 years**				
Emotional overeating	−1.04 (−3.27, 1.19)	0.36	−0.24 (−2.47, 2)	0.83
Enjoyment of food	2.99 (0.76, 5.23)	<0.01	**2.73 (0.51, 4.91)**	**0.02**
Food responsiveness	2.39 (0.17, 4.61)	0.04	**3.12 (0.91, 5.32)**	**<0.01**
**At 10 years**				
Emotional overeating	0.01 (−2.17, 2.19)	0.99	0.65 (−1.51, 2.81)	0.56
Enjoyment of food	4.01 (1.87, 6.16)	<0.01	**4.01 (1.87, 6.16)**	**<0.01**
Food responsiveness	2.67 (0.51, 4.82)	0.02	**3.11 (0.97, 5.25)**	**<0.01**
**Subcortical gray matter volume at 13 years^a^ (cm^**3**^)**				
**At 4 years**				
Emotional overeating	−0.2 (−0.42, 0.01)	0.06	−0.12 (−0.33, 0.09)	0.27
Enjoyment of food	0.27 (0.06, 0.48)	0.01	**0.24 (0.03, 0.45)**	**0.03**
Food responsiveness	0.21 (0.00, 0.42)	<0.05	**0.29 (0.08, 0.50)**	**<0.01**
**At 10 years**				
Emotional overeating	0.01 (−0.2, 0.22)	0.93	0.07 (−0.13, 0.28)	0.49
Enjoyment of food	0.36 (0.16, 0.57)	<0.01	**0.30 (0.10, 0.51)**	**<0.01**
Food responsiveness	0.19 (−0.01, 0.40)	0.07	**0.24 (0.03, 0.44)**	**0.02**

*β coefficients and 95% confidence intervals (CI) are from multiple linear regression. The effect estimates represent the difference in cubic centimeters of brain volumes per 1 SD increase of food-approach eating behaviors. Model 1 was adjusted for child sex, age at the MRI measurement. Model 2 was additionally adjusted for child national origin, energy intake, maternal education, household income, maternal smoking during pregnancy, and maternal prenatal psychopathology symptoms*.

*Statistical significance after multiple testing correction using the Benjamini-Hochberg procedure with an FDR ≤ 0.05 is indicated in bold. Correction for multiple testing was performed based on Model 2*.

a *Additionally adjusted for intracranial volume in model 2*.

We found no associations of binge-eating symptoms at 13 years with global brain volumes (Table 3). Similarly, no associations of binge-eating symptoms with defined regions of interest were found (Supplementary Table 3).

**Table 3:** Associations of binge eating symptoms at age 13 years with global brain volumes at age 13 years.

**Binge eating symptoms**	**Model 1** **β (95%CI)**	***p***	**Model 2** **β (95%CI)**	***p***
**Cerebral gray matter volume (cm** ^**3**^ **)**				
No	Reference		Reference	
Yes	−0.38 (−11.35, 10.58)	0.95	0.41 (−10.03, 10.84)	0.94
**Cerebral white matter volume (cm** ^**3**^ **)**				
Yes	1.80 (−8.18, 11.78)	0.72	2.50 (−7.34, 12.34)	0.62
**Subcortical gray matter volume (cm** ^**3**^ **)**				
Yes	0.36 (−0.58, 1.30)	0.45	0.41 (−0.52, 1.33)	0.39

*β coefficients and 95% confidence intervals (CI) are from multiple linear regressions. The effect estimates represent the difference in cubic centimeters of brain volumes in children with binge eating symptoms compared to children without the symptoms. Model 1 was adjusted for child sex, and age at the MRI measurement. Model 2 was additionally adjusted for child national origin, energy intake, maternal education, household income, maternal smoking during pregnancy, and maternal prenatal psychopathology symptoms*.

Additional analyses were performed to assess if the associations between food-approach eating behaviors at 10 years and brain volumes at 13 years were independent of diet quality at 8 years or BMI at 6 years. The effect estimates of enjoyment of food and food responsiveness with cerebral white matter and subcortical gray matter remained similar after additionally adjusting for diet quality at age 8 years. However, the associations were no longer statistically significant after adjusting for BMI at age 6 years (Supplementary Table 4).

## Discussion

Our study is, to our knowledge, the first to investigate food-approach eating behaviors with brain morphology in adolescents in a large population-based cohort study. Findings suggest that food responsiveness and enjoyment of food in early and mid-childhood are associated with brain development in adolescence. Contrary to our hypothesis, we found positive associations between these two food-approach eating behaviors and global brain measurements. Emotional overeating in childhood as well as binge-eating symptoms in early adolescence were not significantly associated with adolescents' brain morphology. As there is limited evidence for the association between eating behaviors and brain volumes in youth from a general population so far, future studies are needed to replicate our findings.

Our most consistent findings related to brain morphology are for enjoyment of food and food responsiveness. These behaviors, at both ages 4 and 10 years, were consistently associated with a higher cerebral white matter and subcortical gray matter volume. The consistency across ages can probably be explained by the high stability of eating behaviors across the childhood years, as previously reported by Derks et al. (40) in the Generation R Study. However, the associations with cerebral gray matter volume were only observed for enjoyment of food and food responsiveness at age 4 years, but not at 10 years of age. Forkert et al. (41) suggested that gray matter volume has a non-linear increase from birth, which reaches its peak at ~9 years. However, the brain measurements in our study were collected at age 13 years, when the gray matter development is plateaued or even has slightly decreased already. Furthermore, the positive direction was not in line with our hypotheses, which were based on samples of adults with overweight and obesity or binge-eating disorders in whom a smaller brain volume was observed (23, 24, 26, 42–44), while the adolescents in our study sample are from the general population. Possibly, there might be an interaction between weight status and food-approach eating behaviors on brain development which may explain this inconsistency in findings. As most children in our study population had a healthy weight, the positive relation that we found may be driven by children with a normal weight. Unfortunately, we had low statistical power to test interactions with weight status, particularly because of the low number of children with overweight or obesity. Another explanation for the inconsistency between our and previous findings may lie in the observation of a cohort study showing that higher levels of enjoyment of food among children with a normal weight were associated with higher vegetable and fruit liking (12). This may result in a better food quality, which in turn can sustain a better brain growth and overall mental health (5, 45).

The findings of our additional analyses suggested that the associations of enjoyment of food and food responsiveness at 10 years with brain volumes at 13 years are not independent of children's BMI at 6 years. This may not be surprising, considering the findings from previous work on the direction of the association of child BMI and eating behaviors in the same cohort study (46). The study found that BMI at pre-school age predicted more food approach eating behaviors at the age of 10 years rather than reverse, indicating that children with high BMI at young ages may have an up-regulation in appetite. Also, BMI has been linked to brain morphological differences in several studies in children (20, 47). Taken together, although BMI may be a confounder in the association of food-approach eating behaviors and brain morphology, higher BMI may also co-occur with higher levels of food-approach eating behaviors to drive the association.

We found no statistically significant results when examining brain morphology and emotional overeating. Although not statistically significant, at the age of 4 years, emotional eating behavior was negatively associated with all global brain measurements, whereas these associations became positive at the age of 10 years. A previous study working using data from the same cohort suggested that this specific food-approach eating behavior develops in later childhood, as it seems a learned behavior and therefore is influenced by environmental factors (40). Furthermore, the self-assessed binge-type eating symptoms that we used in the study can only indicate a potential subclinical state of binge-eating disorder in participants. Nevertheless, our strict distinction of frequent and non-frequent binge-type symptoms simulated the worldwide prevalence of binge-eating disorder, with 5.7% participants in our cohort having frequent symptoms as compared to a 2.6% lifetime prevalence in a US cohort (48). Many clinical studies have included binge-eating disorder patients, and pointed at an increased volume in the areas of the processing of basic sensory information of food (insular cortex, right frontal operculum, and orbitofrontal cortex) as characteristics for the clinical diagnosis of this eating disorder (49). Our study sample with frequent binge-type symptoms showed this direction of association too, but results were not statistically significant, probably in part due to the small sample size and thus lack of power. This may also apply to emotional overeating, which was assessed on a continuous scale in our study sample but with only few participants showing very high levels of emotional overeating. In sum, participants in previous studies most often had a chronical state of obesity or disordered eating behaviors, which does not match with our study characteristics, where the assessments of eating behaviors may only represent the presence of a certain developmental phase rather than distinguishing clinical symptoms. Indeed, our study is a population-based and not a clinical sample, which on the one hand introduces greater heterogeneity, on the other hand increases the generalizability of the findings. Such studies can generate important information and we encourage future studies to investigate in large population-based samples the direction and causality of associations.

### Strengths and Limitations

The strengths of our study include the large, prospective, population-based design with repeated measures of food-approach eating behaviors. In addition, we were able to integrate a comprehensive assessment of various important parental and child variables as potential confounders. There were also some limitations to consider. First, the CEBQ was reported by the parents, most often the mother of the child. Maternal ratings can influence the perception and rating of the child's eating behavior due to a mother's own beliefs about eating and weight status of the child. Preferably, child behaviors are measured with a multi-informant strategy or by a combination of parent reports and objective measures. Yet, a validation study found moderate associations between children's behavioral measures of eating and parental reported CEBQ (12), supporting that mothers can accurately report eating behaviors of their children. Second, food-approach eating behaviors were assessed only twice and binge eating only once. Greater frequency of assessments with closer time points in this developmental period of life can help determine individual variability and developmental differences in these measures. For instance, using several measurement points of the CEBQ might help detecting a potential non-linearity of the behavior patterns. On the other hand, comparing eating behaviors and brain measurements at a different time point can be also seen as a limitation of the study. Lastly, non-dutch adolescents with lower socioeconomic backgrounds were relatively often lost to follow-up (50), which could influence the generalizability of findings. However, a considerable number of non-dutch families continued to participate in our study.

## Conclusion

Childhood and adolescence are critical periods in life for brain development. Understanding changes of the brain structure during this period of life allows identifying neurophysiological mechanisms and can help to establish environmental influences to prevent those alterations. Examining the relationship of food-approach eating behaviors and brain morphology showed that food responsiveness and enjoyment of food in childhood were associated with brain development in early adolescence. However, despite the temporal order, we cannot conclude that food-approach eating behaviors affected brain development, as it might also be reversed. Future studies are needed to assess the causality as well as the neurobiological mechanisms of each food-approach eating behavior individually in a pediatric population with a follow-up.

## Data Availability Statement

Data described in the manuscript, code book, and analytic code can be made available upon request to Datamanagementgenr@erasmusmc.nl and will be discussed in the Generation R Study Management Team.

## Ethics Statement

The studies involving human participants were reviewed and approved by Erasmus University Medical Center, Rotterdam. Written informed consent to participate in this study was provided by the participants' legal guardian/next of kin.

## Author Contributions

OD, YM, TV, TW, and PJ contributed to conception and design of the study. TW organized the database. OD and YM performed the statistical analysis and wrote the first draft of the manuscript. All authors contributed to manuscript revision, read, and approved the submitted version.

## Funding

The general design of the Generation R Study is made possible by financial support from the Erasmus University Medical Center, Rotterdam, Erasmus University Rotterdam, the Netherlands Organization for Health Research and Development (ZonMw), Netherlands Organization for Scientific Research (NWO), Ministry of Health, Welfare and Sport and Ministry of Youth and Families. YM was supported by China Scholarship Council (CSC) Ph.D. Fellowship for her Ph.D. study in Erasmus Medical Center, Rotterdam, Netherlands. The scholarship file number is 201806240125, CSC URL: http://www.csc.edu.cn/. PJ was supported by a grant from ZonMw (Mental Health Care Research Program - Fellowship 636320005). The neuroimaging data collection and image processing was supported by TOP Grant 91211021 to TW. Supercomputing resources were supported by the NWO Physical Sciences Division (Exacte Wetenschappen) and SURFsara (Cartesius compute cluster, www.surfsara.nl). The funders had no role in the study design, data collection, management, analysis and interpretation of data, and preparation or writing the manuscript. The publication of the study was covered by Nestlé Institute of Health Sciences, Nestlé Research Lausanne, Switzerland.

## Conflict of Interest

The authors declare that the research was conducted in the absence of any commercial or financial relationships that could be construed as a potential conflict of interest.

## Publisher's Note

All claims expressed in this article are solely those of the authors and do not necessarily represent those of their affiliated organizations, or those of the publisher, the editors and the reviewers. Any product that may be evaluated in this article, or claim that may be made by its manufacturer, is not guaranteed or endorsed by the publisher.
