# Supplementary material for: Food-Approach Eating Behaviors and Brain Morphology: The Generation R Study
Source: Front Nutr. 2022 Apr 4;9:846148. doi: 10.3389/fnut.2022.846148 (PMC9014090; doi:10.3389/fnut.2022.846148)
Supplement: Supplementary file 1 [file Data_Sheet_1.docx]

Supplemental materials

**Food-approach eating behaviors and brain morphology: the Generation R Study**

**Olga Dmitrichenko^1,2,3,4^**† **, Yuchan Mou^1,2^** †**, Trudy Voortman^1,4^, Tonya White ^6,7*+^, Pauline W Jansen ^6,8+^**

† *these authors contributed equally to this work and share first authorship*

*+ these authors contributed equally to this work and share last authorship*

^1^ Department of Epidemiology, Erasmus University Medical Center, Rotterdam, The Netherlands.

^2^ The Generation R Study Group, Erasmus University Medical Center, Rotterdam, The Netherlands.

^3^ Institute for Medical Information Processing, Biometry and Epidemiology, Ludwig-Maximilians-Universität München, Munich, Germany.

^4^ Pettenkofer School of Public Health, Munich, Germany.

^5^ Division of Human Nutrition and Health, Wageningen University & Research, Wageningen, The Netherlands.

^6^ Department of Child and Adolescent Psychiatry/Psychology, Erasmus University Medical Center, Rotterdam, The Netherlands.

^7^ Department of Radiology, Erasmus University Medical Center, Rotterdam, The Netherlands.

^8^ Department of Psychology, Education and Child Studies, Erasmus University Rotterdam, Rotterdam, The Netherlands.

*** Correspondence to:**Tonya White
t.white@erasmusmc.nl

**Supplemental Table 1**: General characteristics of all eating behaviors

| **Characteristics** | N | Mean (SD)1 or N (%) | Range for continuous variables | Standardized score,  median [SD]^2^ |
| --- | --- | --- | --- | --- |
| **CEBQ^2^  at 4 years questionnaire, mean (SD)** |  |  |  |  |
| Emotional Overeating | 1526 | 6.0 (2.7) | [4, 20] | -0.7 [1.7] |
| Enjoyment of Food | 1536 | 13.5 (2.9) | [4, 20] | 0.17 [1.4] |
| Food Responsiveness | 1546 | 8.9 (3.4) | [5, 25] | -0.3 [1.18] |
| **CEBQ at 10 years questionnaire, mean (SD)** |  |  |  |  |
| Emotional Overeating | 1568 | 6.0 (2.7) | [4, 20] | -0.7 [1.5] |
| Enjoyment of Food | 1607 | 14.4 (2.7) | [4, 20] | 0.2 [1.5] |
| Food Responsiveness | 1607 | 9.2 (3.8) | [5, 25] | -0.3 [1.3] |
| **Presence of binge-eating symptoms** |  | 74 (5.7%) | - |  |

**Supplemental Table 2:** Post-hoc analysis for food-approach eating behaviors at 4 and 10 years

| **Food-approach eating behaviors (per SD)** | Model 1  *β (95%CI)* | | *p* | Model 2  *β (95%CI)* | | | *p* |
| --- | --- | --- | --- | --- | --- | --- | --- |
|  | **Amygdala (mm^3^)** | | | | | | |
| ***CEBQ at 4 years*** |  | |  |  | | |  |
| Emotional overeating | -4.94  (-13.89, 4.00) | | 0.28 | -3.11  (-12.08, 5.86) | | | 0.5 |
| Enjoyment of food | 8.66  (-0.27, 17.59) | | 0.06 | 7.86  (-1.08, 16.8) | | | 0.08 |
| Food responsiveness | 10.43  (1.55, 19.31) | | 0.02 | 12.32  (3.45, 21.20) | | | <0.01 |
| ***CEBQ at 10 years*** |  | |  |  | | |  |
| Emotional overeating | -1.57  (-10.40, 7.25) | | 0.73 | 0.48  (-8.34, 9.29) | | | 0.92 |
| Enjoyment of food | 10.41  (1.70, 19.11) | | 0.02 | 8.57  (-0.11, 17.24) | | | 0.05 |
| Food responsiveness | 8.13  (-0.27, 17.59) | | 0.06 | 7.86  (-1.08, 16.80) | | | 0.08 |
|  | **Hippocampus (mm^3^)** | | | | | | |
| ***CEBQ at 4 years*** | | | | | | | |
| Emotional overeating | -21.21  (-39.00, -3.41) | 0.02 | | | -16.6  (-34.36, 1.16) | 0.07 | |
| Enjoyment of food | 11.32  (-6.55, 29.18) | 0.21 | | | 9.73  (-8.11, 27.57) | 0.28 | |
| Food responsiveness | 17.05  (-0.67, 34.77) | 0.06 | | | 21.62  (3.99, 39.25) | 0.02 | |
| ***CEBQ at 10 years*** |  |  | | |  |  | |
| Emotional overeating | -5.91  (-23.37, 11.56) | 0.5 | | | -2.02  (-19.42, 15.38) | 0.82 | |
| Enjoyment of food | 13.32  (-3.88, 30.52) | 0.13 | | | 9.23  (-7.88. 26.34) | 0.29 | |
| Food responsiveness | 8.76  (-8.43, 25.96) | 0.32 | | | 11.17  (-5.95, 28.29) | 0.20 | |
| β coefficients and 95% confidence intervals (CI) are from multiple linear regression. The effect estimates represent the difference in cubic millimeters of brain volumes per 1 SD increase of food-approach eating behaviors. Model 1 was adjusted for child sex, age at the MRI measurement. Model 2 was additionally adjusted for child national origin, energy intake, maternal education, household income, maternal smoking during pregnancy, maternal prenatal psychopathology symptoms and intracranial volume.  Statistical significance after multiple testing correction using the Benjamini-Hochberg procedure with an FDR ≤ 0.05 is indicated in **bold**. Correction for multiple testing was performed based on Model 2. | | | | | | | |

**Supplemental Table 3**: Association of binge-type eating symptoms and regions of interest at the age of 13 years

|  | Model 1 |  | Model 2 |  |
| --- | --- | --- | --- | --- |
|  | *β (95%CI)* | *p* | *β (95%CI)* | *p* |
| Insular cortex | 149.6  (-229.7, 528.9) | 0.44 | 184.7  (-185.5, 5541.91) | 0.33 |
| Orbitofrontal cortex | -134.6  (-830.1, 560.93) | 0.70 | -79.69  (-759.14, 599.76) | 0.82 |
| Right frontal operculum | -53.98  (-233.33, 125.37) | 0.56 | -44.3  (-221.5, 132.9) | 0.62 |
| β coefficients and 95% confidence intervals (CI) are from multiple linear regression. The effect estimates represent the difference in cubic centimeters of brain volumes per 1 SD increase of food-approach eating behaviors. Model 1 was adjusted for child sex, age at the MRI measurement. Model 2 was additionally adjusted for child national origin, energy intake, maternal education, household income, maternal smoking during pregnancy, and maternal prenatal psychopathology symptoms.  Statistical significance after multiple testing correction using the Benjamini-Hochberg procedure with an FDR ≤ 0.05 is indicated in **bold**. Correction for multiple testing was performed based on Model 2. | | | | |

**Supplemental Table 4**: Additional analyses of association between children’s food-approach eating behaviors at 10 years and brain volumes at 13 years of age

| **Food-approach eating behaviors (per SD)** | Model 1  *β (95%CI)* | *p* | | Model 2  *β (95%CI)* | *p* | |  |  |
| --- | --- | --- | --- | --- | --- | --- | --- | --- |
|  | **Cerebral gray matter volume (cm^3^)** | | | | | |  |  |
| ***CEBQ at 10 years*** |  |  | |  |  | |  |  |
| Emotional overeating | 0.41  (-1.33, 3.29) | 0.98 | | 0.08  (-2.24, 2.39) | 0.95 | |  |  |
| Enjoyment of food | 1.41  (-0.91, 3.73) | 0.23 | | 0.71  (-1.6, 3.01) | 0.55 | |  |  |
| Food responsiveness | 0.73  (-1.55,3.02) | 0.53 | | -1.9  (-4.4, 0.59) | 0.13 | |  |  |
|  | **Cerebral white matter volume (cm^3^)** | | | | | | |  |
| ***CEBQ at 10 years*** |  | |  |  | |  | | |
| Emotional overeating | 0.68  (-1.49, 2.85) | | 0.54 | -0.19  (-2.37, 1.98) | | 0.86 | | |
| Enjoyment of food | **3.16**  **(0.99, 5.34)** | | **<0.01** | 2.36  (0.2, 4.52) | | 0.03 | | |
| Food responsiveness | **2.92**  **(0.76, 5.08)** | | **0.01** | 0.71  (-1.65, 3.08) | | 0.55 | | |
|  | **Subcortical gray matter volume (cm^3^)** ^1^ | | | | | | |  |
| ***CEBQ at 10 years*** |  | |  |  | |  | | |
| Emotional overeating | 0.07  (-0.13, 0.28) | | 0.07 | -0.01  (-0.23, 0.21) | | 0.92 | | |
| Enjoyment of food | **0.27**  **(0.06, 0.47)** | | **0.01** | 0.19  (-0.01, 0.4) | | 0.07 | | |
| Food responsiveness | 0.21  (0.01, 0.42) | | 0.04 | -0.01  (-0.23, 0.21) | | 0.93 | | |
| β coefficients and 95% confidence intervals (CI) are from multiple linear regression. The effect estimates represent the difference in cubic centimeters of brain volumes per 1 SD increase of food-approach eating behaviors. Model 1 was adjusted for child sex, age at the MRI measurement, child national origin, energy intake, maternal education, household income, maternal smoking during pregnancy, maternal prenatal psychopathology symptoms and **diet quality score at 8 years**. Model 2 was adjusted for child sex, age at the MRI measurement, child national origin, energy intake, maternal education, household income, maternal smoking during pregnancy, maternal prenatal psychopathology symptoms and **child BMI at 6 years**  Statistical significance after multiple testing correction using the Benjamini-Hochberg procedure with an FDR ≤ 0.05 is indicated in **bold**. Correction for multiple testing was performed based on Model 2.  ^1^ Additionally adjusted for intracranial volume in model 2. | | | | | | | |  |

**Supplemental Figure 1:** Flowchart of study population

Children with food-approach eating behaviors data at 4 or 10 years

N=5536

Children with consent for participation in the postnatal phase

N=6625

Excluded children without food-approach eating behaviors data at 4 or 10 years

N=1089

N=1089

Excluded children who had

- No MRI consent (N=2743)
- Imaging failed to be reconstructed using FreeSurfer (N=1003)
- Major incidental findings (N=9)

N=3755

1781 children with both food-approach eating behavior data and MRI data were included as the study population

Children with a CEBQ at 10 and an evaluable MRI scan at 13 years

N=1642

Children with a CEBQ at 4 and an evaluable MRI scan at 13 years

N=1559
